# Achieving sequential therapy in advanced gastric cancer: the importance of appropriate patient management for the elderly and/or those with ascites

**DOI:** 10.1007/s10120-020-01067-3

**Published:** 2020-04-01

**Authors:** Yasuo Hamamoto, Yongzhe Piao, Akitaka Makiyama

**Affiliations:** 1 Keio Cancer Center, Keio University School of Medicine, Tokyo, Japan; 2 Eli Lilly Japan K.K., Kobe, Japan; 3 Cancer Center, Gifu University Hospital, 1-1 Yanagido, Gifu, 501-1194 Japan

**Keywords:** Gastric cancer, Elderly patient, Ascites, Sequential therapy

## Abstract

Treatment options for patients with advanced gastric cancer (AGC) are limited. One approach to improving survival in patients with AGC is to optimize the available agents via sequential therapy. However, clinical trial reports of first-line chemotherapy indicate that elderly patients and patients with massive ascites are less likely to receive subsequent lines of therapy. In addition, clinical trials of second- and third-line chemotherapy generally exclude these two patient populations because they are likely to have poor performance status and additional issues that are difficult to manage. Good patient management is likely to be key to the successful use of sequential therapy in these two patient populations by minimizing adverse effects to allow patients to derive benefit from the additional treatment. This narrative review summarizes the available information on AGC treatment and patient management in elderly patients and patients with massive ascites. The available data suggest that elderly patients benefit from chemotherapy; however, monitoring toxicity is essential to avoid chemotherapy-related toxicities. Important aspects of patient management for elderly patients include symptom monitoring, nutritional support, and fall prevention. The available data for patients with massive ascites show limited success for a range of treatment approaches, including systemic chemotherapy. The management of ascites is also challenging, with no clear guidance on the preferred strategies. To address these gaps in knowledge, future clinical trials should incorporate more inclusive eligibility criteria to enroll populations of patients with AGC that are more reflective of the real-world population with respect to age, complications, and overall health status.

## Introduction

Gastric cancer is the fifth most frequently diagnosed cancer and the third leading cause of cancer mortality worldwide [1]. Chemotherapy remains the main treatment option for patients with advanced gastric cancer (AGC), with Japanese, US, and European treatment guidelines recommending chemotherapy as combination therapy or monotherapy from the first-line setting through to second- and later-line settings [2–4]. However, there are few targeted therapies available for patients with AGC. Trastuzumab in combination with chemotherapy is used as first-line treatment for HER2-positive AGC [5], ramucirumab is used as a second-line treatment for AGC as monotherapy or in combination with paclitaxel [6, 7], and nivolumab for third- or later-line treatment [8]. Despite these targeted therapies, treatment options for AGC are still very limited and patient survival is poor [9]. Therefore, it is critical that the benefit of current treatment options is optimized as far as possible.

**Fig. 1:**
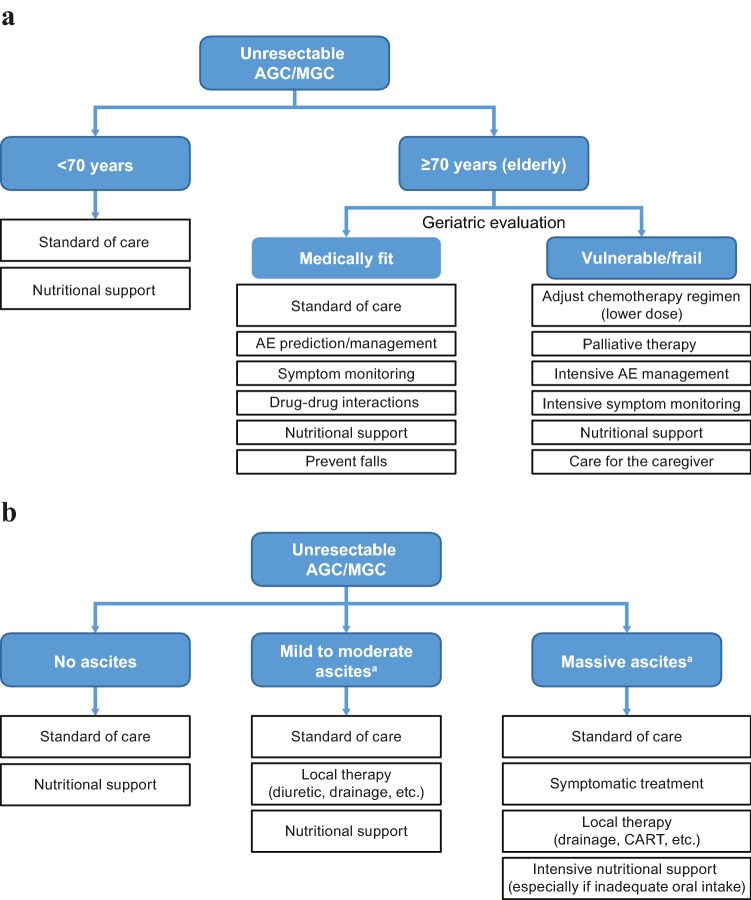
Management of subsets of patients with AGC with respect to age (**a**) and ascites (**b**). *AE* adverse event, *AGC* advanced gastric cancer, *CART* concentrated ascites reinfusion therapy, *MGC* metastatic gastric cancer. ^a^If nonmeasurable lesions only: proper and timely identification of disease progression based on evaluation of combined radiographic images, symptoms, and tumor markers

One strategy that may improve the benefit of existing treatments for AGC is sequential therapy, which allows patients to receive multiple treatments with different mechanisms of action, ie, by expanding the lines of treatment from first-line to second-line and beyond [10]. Findings from several meta-analyses suggest that inclusion of multiple lines of therapy can improve survival in patients with AGC [8] and that third-line therapy (chemotherapy or targeted therapy) improves survival compared with placebo/best supportive care [11]. In Japan, sequential therapy is more frequently incorporated into the treatment paradigm, and this has been associated with increased overall survival (OS) and longer postprogression survival compared with studies conducted in the rest of the world [12]. Meta-analyses revealed that there were strong correlations between receiving second-line chemotherapy and OS, as well as postprogression survival in Japanese patients enrolled in phase 3 clinical trials. The analyses also suggested that other factors such as third-line chemotherapy may have also contributed to prolonged postprogression survival [13]. Although studies assessing why patients with AGC might not receive subsequent lines of treatment after failure of first-line treatment are lacking, performance status (PS) appears to be an important factor. Because of poor PS, elderly patients and patients with massive ascites may not be considered for a subsequent line(s) of treatment, particularly because these patients have decreased organ function and comorbidities and, therefore, are at greater risk of toxicity and complications [14–18]. Good patient management will likely play an important role in sequential therapy by minimizing the adverse effects of treatment while maximizing the benefits. However, elderly patients and patients with massive ascites may have additional issues that are difficult to manage and require special attention (Fig. 1). This review discusses the management of elderly patients and patients with ascites separately. We recognize that ascites may occur in elderly patients with AGC, in which case a patient management strategy combining aspects of the individual management strategies discussed here should be employed.

## Management of elderly patients with advanced gastric cancer

As a result of prolonged life expectancy and aging of the world’s population, the number of elderly patients with AGC is expected to increase substantially. However, elderly patients with cancer are less likely to receive recommended treatments because of the higher risk of toxicity owing to their higher incidence of comorbidities, age-related changes in pharmacokinetics and pharmacodynamics, concomitant medications, and decreased major organ function [19, 20]. It is important to note that there is no standard definition of “elderly” [19, 21] and although many organizations, including the World Health Organization, define the elderly as those 65 years and over, increased life expectancy is changing this definition [21]. As such, 65, 70, 75, and 80 years are used as the threshold for elderly in the evaluation of gastric cancer treatment [19]. In addition, because aging is an individual process, chronological age may differ from biological age and, therefore, chronological age alone is not necessarily a predictor of physiological fitness [22, 23].

### Clinical trial data

Clinical trial data that are available regarding the treatment of elderly patients with AGC is limited and is mostly derived from phase II studies conducted in elderly patients, age subgroup analyses of phase III studies, and retrospective analyses of elderly patient populations. Overall, evidence from these clinical trials suggests that chemotherapy is feasible in elderly patients with AGC [14, 24–28], but elderly patients with AGC are less likely to receive multiple lines of chemotherapy than younger patients [14, 15]. For example, studies of first-line chemotherapy in elderly vs younger patients have reported rates of second-line chemotherapy of 38.7% vs 62.7% [14] and 24% vs 68% [15]. Taken together, the available clinical trial data suggest that age alone is not a barrier to receiving treatment for AGC; elderly patients can derive benefit from chemotherapy, but toxicity monitoring is essential to minimize the onset of chemotherapy-related toxicities (Fig. 1a).

### Patient management

#### Geriatric assessment

The aging process varies widely among individuals; elderly people of the same age do not necessarily have similar physical conditions, so treatment should be guided by individual health status. However, traditional oncology measures of PS, such as the Eastern Cooperative Oncology Group (ECOG) scale and Karnofsky Performance Scale, may not be reliable for assessing physical status in elderly patients because they do not take into account comorbidities or other aspects of frailty [29, 30]. Consequently, performing a comprehensive geriatric assessment (CGA) is recommended for predicting chemotherapy toxicity and mortality in elderly patients with cancer (Fig. 1a) [31]. A CGA is a series of standardized tests evaluating aspects of physical performance, comorbidity, cognition, medications, nutritional status, functional status, mental health, and social status in elderly patients [23]. Conducting a CGA can help guide treatment decisions based on the overall health status of the elderly patient, preventing the undertreatment of medically fit elderly patients as well as limiting treatment intensity in medically unfit elderly patients [23]. It should be noted that the CGA studies described below were conducted in patients with various tumor types; therefore, the results may not be directly applicable to patients with AGC.

A potential role for CGA may be to help predict which elderly patients are at risk of experiencing chemotherapy-related toxicities or not completing treatment (Fig. 1a). Three items on the Mini Nutrition Assessment (MNA; psychological distress or acute disease in past 3 months, neuropsychological problems, and using > 3 prescription drugs) independently predicted premature discontinuation of chemotherapy [32]. In a comparative study of elderly patients with various cancers (approximately half with gastrointestinal cancer), those whose treatment was based on a CGA were more likely to complete cancer treatment as planned and had fewer treatment modifications than those who received routine care; however, grade ≥ 3 toxicity and 6-month mortality did not differ between groups [33]. In the randomized phase III ESOGIA study, treatment allocation on the basis of CGA resulted in significantly less all-grade toxicity (but not grade 3/4 toxicity) and significantly fewer treatment failures due to toxicity compared with treatment allocation on the basis of PS and age in elderly patients with advanced NSCLC [34]. The ability of CGA to predict chemotherapy-related toxicities has been used to guide the choice of treatment regimen, with a systematic review of 35 studies showing that, after CGA, treatment plans were modified in 28% of patients, usually to a less intensive regimen.

Other scoring systems have been designed to identify patients at risk of chemotherapy-related toxicities. The CRASH (Chemotherapy Risk Assessment Scale for High-Age Patients) score categorizes patients into different risk categories for toxicities according to diastolic blood pressure, IADL, lactate dehydrogenase levels, toxicity of individual chemotherapy drugs, ECOG PS, Mini-Mental Health Status, and MNA [35]. The Cancer and Aging Research Group (CARG) tool model includes age, cancer type (with gastrointestinal or genitourinary cancer having a higher risk score), chemotherapy dosing, number of chemotherapy drugs, laboratory measures, hearing level, walking ability, number of recent falls, IADL score, and social activity level [29, 36].

A related aspect to consider is the concept of frailty, defined as a complex, multidimensional, and cyclical state of diminished physiological reserve that results in decreased resiliency and adaptive capacity, and increased vulnerability to stressors [30]. Frail patients are at increased risk of intolerance to chemotherapy, disease progression, and death; therefore, additional aspects of care are required (Fig. 1a) [30]. Over 70 different measures of frailty have been proposed, including the CGA; however, the optimal measure for screening and assessment of frailty is unclear [30].

Incorporation of CGA in prospective clinical trials of elderly patients with AGC, in particular, patients with poor PS, would help guide treatment choice in this population. Further research is required to determine which CGA items are most useful for predicting survival and toxicities in patients with AGC.

#### Symptom monitoring

Elderly patients with cancer may have symptoms such as fatigue, decreased appetite, pain, nausea, and depression arising from the cancer and/or treatment [37, 38]. Elderly patients may underreport symptoms for several reasons, including viewing them as a normal part of aging, concern that the oncologist may discontinue treatment, and cognitive impairment and depression [37–39]. Additionally, elderly patients may forget to report adverse effects occurring early in the chemotherapy cycle that have since resolved [39]. Identifying toxicities early in the treatment cycle would allow the oncologist to intervene by adding/increasing supportive medications, modifying the chemotherapy dose, and/or referring the patient to a palliative care provider, which may reduce hospitalizations and emergency department visits (Fig. 1a) [39].

Numerous scales and questionnaires are available to measure the various symptoms associated with cancer and its treatment [37, 38]. More recently, a number of computer-/tablet-based patient-reported outcome (PRO) systems have been developed and shown to be feasible for use in the clinical care setting (e.g., Symptom Tracking and Reporting [STAR], Advanced Symptom Management System in Palliative Care [ASyMSp]) [39, 40]. Basch et al. [41] found that web-based symptom reporting resulted in a better quality of life (QoL), fewer emergency department visits, fewer hospitalizations, longer duration of palliative chemotherapy, and better quality-adjusted survival in patients with advanced solid tumors. Denis et al. [42] found that web-mediated follow-up improved OS because of early relapse detection, with better PS at relapse, in patients with advanced-stage lung cancer. Although these two studies included elderly patients, studies to investigate if elderly patients derive the same benefits from electronic PRO systems as younger patients are needed [39].

#### Polypharmacy and drug-drug interactions

Elderly patients with cancer are likely to experience polypharmacy (e.g., concurrent use of ≥ 5 prescription/over-the-counter medications and herbal supplements [43]). In addition to receiving cancer therapy and supportive medications to prevent side effects, elderly patients are likely to have comorbidities requiring drug therapy. A complex medication regimen can be difficult to manage, potentially leading to inappropriate medication use and nonadherence, with a risk of increased and overlapping side effects [44, 45].

Although polypharmacy does not necessarily result in inappropriate drug combinations, there is a risk of drug-drug interactions (DDIs), which is increased in elderly patients with cancer because of age- and comorbidity-related changes in drug absorption and excretion [44, 46]. DDIs are an important concern (Fig. 1a) because of the potential to affect drug dosage, resulting in reduced efficacy or excessive toxicity [46]. Of note, because the metabolism, distribution, and elimination of monoclonal antibodies are not mediated by cytochrome P450 or drug transporters, monoclonal antibody therapies are not expected to compete directly with chemically-derived drugs and, therefore, the risk of DDIs may be lower with such therapies [47, 48]. In 13 studies of elderly patients with cancer, DDI prevalences ranging from 2 to 77% were reported [43]. However, it should be noted that the studies differed in trial design, methodology, and, importantly, definitions of DDIs. To avoid harmful DDIs, the following are strongly recommended: a routine reassessment of all prescription and over-the-counter medications and herbal supplements; communication of a complete list of medications between healthcare providers on every referral, hospital admission, hospital transfer, and hospital discharge; and monitoring for signs of DDIs [46, 49, 50]. However, the optimal approach for screening for DDIs (e.g., comprehensive medication review by a clinical pharmacist, consultation with a clinical pharmacologist, use of clinical decision support software) remains to be determined [43].

#### Nutritional support

Nutritional status is of great importance during the treatment of all patients with cancer, and particularly in the elderly (Fig. 1a). Poor nutritional status/malnutrition is associated with increased risk of toxicity from chemotherapy and worse survival outcomes [51, 52] and, in the elderly, inadequate protein intake can result in a reduction in lean muscle mass and increased risk of frailty [53]. Malnourished patients commonly need dose reductions/delays, and possibly treatment discontinuation, and have a higher frequency of hospitalization, reduced QoL, and decreased survival [10]. The International Society for Geriatric Oncology recommends including nutritional assessment before starting cancer treatment [51]. Among the available nutritional assessment tools, the MNA Short Form was designed for elderly patients with cancer and includes questions on food intake, weight history, mobility, acute disease, neuropsychological problems, and body mass index [51, 52]. However, nutritional assessment tools may not detect other symptoms that affect nutrition such as nausea and vomiting, which are common symptoms in patients with AGC receiving chemotherapy [52].

Nutritional support may be of benefit in patients with AGC with respect to QoL and survival [10] and, as recommended by the American Society for Enhanced Recovery and Perioperative Quality Initiative Joint Consensus Statement, is an important contributor to recovery in elderly patients who have undergone major surgery, especially surgery for AGC [53]. Following surgery, elderly patients can be at risk of malnutrition for a range of factors including decreased appetite, adverse effects of analgesics, and a lack of awareness of how diet can support recovery [53]. Evidence from a clinical trial of elderly inpatients who were at risk of malnutrition demonstrated a 49% reduction in 90-day mortality and indicators of nutritional status in those receiving high-protein oral supplementation compared with placebo [54]. Despite this, there are no specific guidelines for elderly patients with AGC or ascites, and studies of the value of nutritional counseling in patients undergoing active cancer treatment have only been conducted in younger populations [51].

#### Prevention of falls

Falls are commonly reported in elderly patients with cancer, with several studies reporting a higher prevalence of falls in elderly patients with a cancer diagnosis compared with those without [55, 56]. Side effects of cancer treatment can contribute to the increased fall risk. For example, neurotoxic agents (e.g., taxanes and platinum agents) have been associated with an increased risk of fall-related injuries, likely due to effects manifested as dizziness or orthostatic hypotension [57]. Falls can result in serious injuries, such as fractures and head trauma, as well as a loss of confidence from fear of falling; this may ultimately lead to functional decline, affecting the patient’s ability to live independently [56, 58]. In addition, previous falls are associated with an increased risk of chemotherapy toxicity and poorer survival [29, 59]. Screening for falls in elderly patients is, therefore, strongly recommended, from simply asking the patient if they have had any falls since their last visit to administering gait and balance tests, such as Timed Up and Go or Gait Speed tests [58, 60]. Patients at risk for falls should be offered a multifactorial assessment followed by referral to a primary care provider, geriatric team, or falls clinic (Fig. 1a) [60]. Possible interventions to prevent falls include evaluation of additional risk factors for falls, home safety evaluation, physical therapy referral for strength and balance training, home exercise program, and fall counseling education [60].

#### Caregivers of elderly patients

Another aspect to consider is the QoL of the caregiver of the elderly patient with cancer (Fig. 1a). Much of the day-to-day care of elderly patients is provided by informal caregivers, defined as a relative, partner, or friend who provides essential support, including assisting with ADL, performing medical- and nursing-related tasks, and providing physical and emotional assistance [61, 62]. However, caregiving is associated with a physical, emotional, and financial toll [61]. Kehoe et al. evaluated the relationship between impaired CGA domains of elderly patients with cancer and their caregivers’ QoL [62]. Higher numbers of patient CGA domain impairments were associated with caregiver depression and lower caregiver QoL, with impaired patient nutrition associated with caregiver depression and impaired patient function associated with lower caregiver QoL. Thus, the CGA potentially provides valuable information on the well-being of the caregiver as well as that of the patient. Equally, patient needs identified by the CGA may ease the burden of the caregiver [61]. For example, patients identified by the CGA as needing assistance with ADL could be referred for nurse/social work evaluations; patients identified as having depression or anxiety could be referred for a psychology/psychiatry evaluation; and patients identified as having unintentional weight loss could be referred for a nutritional consultation [61]. Identifying the needs of the elderly patient early in the treatment course and providing the appropriate assistance would increase support for both patient and caregiver.

## Management of patients with gastric cancer and ascites

Ascites and peritoneal metastases, which are poor prognostic factors in AGC [63, 64], are among the most common complications of AGC. Nearly half of all patients with peritoneal metastases have ascites [65] and, because of their poor PS and concerns over adverse treatment effects, patients with ascites are generally excluded from clinical trials, including the pivotal phase III gastric cancer studies [66, 67]. Moreover, factors associated with massive ascites may limit treatment options. For example, the intolerable abdominal fullness associated with massive ascites makes it difficult to administer cisplatin-based chemotherapy, which requires handling of large fluid loads to achieve adequate hydration for renal protection from cisplatin [67]. In addition, chemotherapeutic agents may be distributed in the ascitic fluid [68, 69], in which case repeated drainage of ascitic fluid can reduce the effective delivery of anticancer therapy.

### Clinical trial data

In general, patients with AGC with ascites and peritoneal metastases are treated with systemic chemotherapy similar to patients with other metastases (Fig. 1b) [66]. Numerous regimens have been investigated, with median survival times of patients in mostly small single-arm studies of systemic chemotherapy ranging between 5.2 and 18 months [66]. Clinical trial data from recent retrospective analyses have shown improvements in ascites following systemic chemotherapy in patients with AGC, including in patients with massive ascites [17, 18, 67, 70]. As patients with massive ascites have few treatment options, are more likely to start treatment on a reduced dose, and may miss the opportunity to receive second-line treatment because of failure of first-line treatment followed by rapid disease progression, it is particularly important to optimize the efficacy of first-line treatment in this patient population [17, 18]. Recently, a phase 2/3 study (JCOG1108/WJOG7312G) of chemotherapy in patients with AGC, severe peritoneal metastases, and massive ascites, reported that sequential treatment was feasible [71]. Approximately 57% of patients went on to receive second-line chemotherapy, and the 1-year OS was ~ 23% [71].

### Patient management

Management of patients with AGC and ascites is challenging; numerous approaches are available with no clear guidance on preferred strategies. Treatment options range from symptomatic relief via drainage procedures to treating underlying cancer with chemotherapy and/or surgery (Fig. 1b). Unfortunately, there are no clearly defined clinical predictors that identify patients who will develop ascites or measures to prevent ascites development. Treatment of ascites should be individualized and take into account survival and QoL issues [72]. Palliative techniques are an important part of managing symptoms and have been directly linked to increasing patient satisfaction with treatment [72].

#### Identification of ascites burden

A key step in the management of ascites is proper and timely identification of peritoneal disease. Cytological assessment of ascitic fluid should occur during the initial evaluation as a positive result is diagnostic, while increased levels of tumor markers such as carcinoembryonic antigen in the ascitic fluid are indicative of accumulating peritoneal fluid from metastatic gastric cancer [73]. Imaging techniques, including ultrasonography and computed tomography (CT), are being evaluated to aid accurate measurement of ascites volumes [73]. CT criteria have been validated in patients with AGC and ascites [73], while no criteria for ultrasonography have been established [73].

In patients with low or no measurable peritoneal lesions, optimal timing for treatment changes is challenging [74]. In these patients, evaluation of disease progression is often achieved using clinical symptoms or tumor markers instead of imaging [74]. Notably, a retrospective observational study reported that treatment modification based on symptoms or tumor markers (carcinoembryonic antigen, carbohydrate antigen 19-9, and CA125) resulted in longer OS compared with treatment changes based on definitive evidence of peritoneal disease determined by CT [74]. Furthermore, the study results suggested that patients whose treatment was modified based on imaging findings appeared to have a higher tumor burden at the time the decision was made to select a second-line therapy than patients whose treatment changes were based on symptoms or tumor markers [74]. Studies have also demonstrated that the inadequate response of ascites volumes to anti-cancer treatment correlated with poor prognosis. Thus, frequent and repetitive clinical and objective assessment of ascites volume, ascites-related symptoms, and tumor markers may be useful in determining when to change treatment [73].

#### Supportive treatments for ascites

In terms of supportive treatment, ascites can be drained by paracentesis, percutaneously implanted catheters or peritoneal ports, and peritoneovenous shunts [75]. A systematic review of drainage techniques reported that symptom control was achieved in approximately 97% of patients after ascites drainage and, importantly, PROs and QoL were also improved after drainage [75]. However, drainage of ascites is associated with fatigue and hemodynamic instability [76], thought to be related to the loss of proteins in the drainage fluid. Cell-free and concentrated ascites reinfusion therapy (CART) has been established in the treatment of patients with ascites from hepatic cirrhosis and is being investigated in patients with malignancy (Fig. 1b) [76]. CART involves filtering ascites to remove cell components (but not vital proteins), concentration to reduce volume during reinfusion (using a plasma-separating membrane), and intravenous reinfusion of the concentrated fluid. Within 24 h of undergoing CART, patients reported that symptoms such as gastric symptoms, respiratory distress, and fatigue were improved [76]. In general, CART is a relatively safe procedure, with AEs limited to fever, hemodynamic changes (associated with paracentesis), and thrombocytopenia [76]. However, care must be taken regarding the free hemoglobin, endotoxin, and heparin that may be added to the original ascites, as these cannot be removed during the CART procedure and will, therefore, be reinfused.

#### Assessing QoL

Management of patients with AGC and ascites should include measurement of symptoms such as anxiety and depression, general well-being, and fatigue (using validated instruments), as well as physical symptoms such as shortness of breath, abdominal distension, and mobility [75]. Successful management of ascites is reliant upon the appropriate selection of drainage technique and starts with a thorough interview of the patient, preferably combined with the use of QoL questionnaires. Recognition of the impact of treatment plans, expected survival, and patient concerns regarding ascites drainage is also important. Ascites drainage is a part of supportive care that should be applied at any time during the disease course, to better control symptoms and improve QoL and treatment outcomes.

#### Nutritional support

An important factor often overlooked in managing ascites is the patient’s nutritional status (Fig. 1b); however, effective methods for measuring nutritional status are lacking. The prognostic nutritional index (PNI), calculated from serum albumin and total lymphocyte counts, may be a useful marker of nutritional status in patients with AGC with peritoneal dissemination and may predict outcomes in this patient population, with malnutrition (defined as a PNI score < 45) associated with significantly shorter OS [77]. This highlights the importance of assessing nutritional status in patients with AGC and peritoneal disease and, more importantly, addressing malnutrition to potentially improve patient survival (Fig. 1b). To this end, the ghrelin receptor agonist anamorelin has been investigated in Japanese patients with advanced gastrointestinal cancer with cachexia, resulting in gains in lean body mass and body weight [78].

## Conclusions

As a result of limited treatment options, AGC still represents a significant unmet medical need. While research continues into appropriate treatment strategies for patients with AGC, there remains much that we can do to improve patient outcomes and QoL, including better monitoring and diagnosis, use of supportive treatments, measuring and treating malnutrition, and supporting patients’ caregivers. Until effective treatments for AGC are identified, a comprehensive approach incorporating these aspects of care is warranted, in conjunction with optimizing the available agents via sequential therapy, utilizing geriatric measures to determine which elderly patients benefit from treatment, and accurately measuring ascites burden in patients with the suspected peritoneal disease.

## References

[1] Bray F, Ferlay J, Soerjomataram I, Siegel RL, Torre LA, Jemal A (2018). Global cancer statistics 2018: GLOBOCAN estimates of incidence and mortality worldwide for 36 cancers in 185 countries. CA Cancer J Clin.

[2] Japanese Gastric Cancer Association (2018) Japanese Gastric Cancer Treatment Guidelines 2018 (ver. 5). Tokyo: Kanehara-Shuppan.

[3] National Comprehensive Cancer Network. Gastric Cancer (Version 2.2019). https://www.nccn.org/professionals/physician_gls/pdf/gastric.pdf. Accessed 4 Oct 2019.

[4] Smyth EC, Verheij M, Allum W, Cunningham D, Cervantes A, Arnold D (2016). Gastric cancer: ESMO Clinical Practice Guidelines for diagnosis, treatment and follow-up. Ann Oncol.

[5] Bang YJ, Van Cutsem E, Feyereislova A, Chung HC, Shen L, Sawaki A (2010). Trastuzumab in combination with chemotherapy versus chemotherapy alone for treatment of HER2-positive advanced gastric or gastro-oesophageal junction cancer (ToGA): a phase 3, open-label, randomised controlled trial. Lancet.

[6] Fuchs CS, Tomasek J, Yong CJ, Dumitru F, Passalacqua R, Goswami C (2014). Ramucirumab monotherapy for previously treated advanced gastric or gastro-oesophageal junction adenocarcinoma (REGARD): an international, randomised, multicentre, placebo-controlled, phase 3 trial. Lancet.

[7] Wilke H, Muro K, Van Cutsem E, Oh SC, Bodoky G, Shimada Y (2014). Ramucirumab plus paclitaxel versus placebo plus paclitaxel in patients with previously treated advanced gastric or gastro-oesophageal junction adenocarcinoma (RAINBOW): a double-blind, randomised phase 3 trial. Lancet Oncol.

[8] Kang YK, Boku N, Satoh T, Ryu MH, Chao Y, Kato K (2017). Nivolumab in patients with advanced gastric or gastro-oesophageal junction cancer refractory to, or intolerant of, at least two previous chemotherapy regimens (ONO-4538-12, ATTRACTION-2): a randomised, double-blind, placebo-controlled, phase 3 trial. Lancet.

[9] Apicella M, Corso S, Giordano S (2017). Targeted therapies for gastric cancer: failures and hopes from clinical trials. Oncotarget.

[10] Salati M, Di Emidio K, Tarantino V, Cascinu S (2017). Second-line treatments: moving towards an opportunity to improve survival in advanced gastric cancer?. ESMO Open.

[11] Zheng Y, Zhu XQ, Ren XG (2017). Third-line chemotherapy in advanced gastric cancer: a systematic review and meta-analysis. Medicine (Baltimore).

[12] Iizumi S, Takashima A, Sakamaki K, Morita S, Boku N (2018). Survival impact of post-progression chemotherapy in advanced gastric cancer: systematic review and meta-analysis. Cancer Chemother Pharmacol.

[13] Takashima A, Iizumi S, Boku N (2017). Survival after failure of first-line chemotherapy in advanced gastric cancer patients: differences between Japan and the rest of the world. Jpn J Clin Oncol.

[14] Cho YH, Kim SY, Hong Lee M, Yoo MW, Bang HY, Lee KY (2012). Comparative analysis of the efficacy and safety of chemotherapy with oxaliplatin plus fluorouracil/leucovorin between elderly patients over 65 years and younger patients with advanced gastric cancer. Gastric Cancer.

[15] Sugimoto A, Nishida T, Osugi N, Takahashi K, Mukai K, Nakamatsu D (2019). Prediction of survival benefit when deciding between chemotherapy and best supportive therapy in elderly patients with advanced gastric cancer: a retrospective cohort study. Mol Clin Oncol.

[16] Iwasa S, Nakajima TE, Nakamura K, Takashima A, Kato K, Hamaguchi T (2012). First-line fluorouracil-based chemotherapy for patients with severe peritoneal disseminated gastric cancer. Gastric Cancer.

[17] Hara H, Kadowaki S, Asayama M, Ooki A, Yamada T, Yoshii T (2018). First-line bolus 5-fluorouracil plus leucovorin for peritoneally disseminated gastric cancer with massive ascites or inadequate oral intake. Int J Clin Oncol.

[18] Matsumoto H, Kawazoe A, Shimada K, Fukuoka S, Kuboki Y, Bando H (2018). A retrospective study of the safety and efficacy of paclitaxel plus ramucirumab in patients with advanced or recurrent gastric cancer with ascites. BMC Cancer.

[19] Saif MW, Makrilia N, Zalonis A, Merikas M, Syrigos K (2010). Gastric cancer in the elderly: an overview. Eur J Surg Oncol.

[20] Kim HS, Kim JH, Kim JW, Kim BC (2016). Chemotherapy in elderly patients with gastric cancer. J Cancer.

[21] Pak LM, Wang J (2017). The appropriate treatment for elderly gastric cancer patients. Art Surg.

[22] Launay-Vacher V, Chatelut E, Lichtman SM, Wildiers H, Steer C, Aapro M (2007). Renal insufficiency in elderly cancer patients: International Society of Geriatric Oncology clinical practice recommendations. Ann Oncol.

[23] Marosi C, Köller M (2016). Challenge of cancer in the elderly. ESMO Open.

[24] Seol YM, Song MK, Choi YJ, Kim GH, Shin HJ, Song GA (2009). Oral fluoropyrimidines (capecitabine or S-1) and cisplatin as first line treatment in elderly patients with advanced gastric cancer: a retrospective study. Jpn J Clin Oncol.

[25] Lim JH, Lee MH, Kim HG, Shin YW, Yi HG, Shin SH (2010). Three-weekly s-1 monotherapy as first-line treatment in elderly patients with recurrent or metastatic gastric cancer. Gut Liver.

[26] Terazawa T, Iwasa S, Takashima A, Nishitani H, Honma Y, Kato K (2013). Impact of adding cisplatin to S-1 in elderly patients with advanced gastric cancer. J Cancer Res Clin Oncol.

[27] Tsushima T, Hironaka S, Boku N, Machida N, Yamazaki K, Yasui H (2013). Comparison of safety and efficacy of S-1 monotherapy and S-1 plus cisplatin therapy in elderly patients with advanced gastric cancer. Int J Clin Oncol.

[28] Makiyama A, Kunieda K, Noguchi M, Kajiwara T, Tamura T, Takeda K (2018). First-line chemotherapy with S-1 alone or S-1 plus cisplatin for elderly patients with advanced gastric cancer: a multicenter propensity score matched study. Gastric Cancer.

[29] Hurria A, Togawa K, Mohile SG, Owusu C, Klepin HD, Gross CP (2011). Predicting chemotherapy toxicity in older adults with cancer: a prospective multicenter study. J Clin Oncol.

[30] Ethun CG, Bilen MA, Jani AB, Maithel SK, Ogan K, Master VA (2017). Frailty and cancer: implications for oncology surgery, medical oncology, and radiation oncology. CA Cancer J Clin.

[31] Wildiers H, Heeren P, Puts M, Topinkova E, Janssen-Heijnen ML, Extermann M (2014). International Society of Geriatric Oncology consensus on geriatric assessment in older patients with cancer. J Clin Oncol.

[32] Aaldriks AA, Maartense E, Nortier HJ, van der Geest LG, le Cessie S, Tanis BC (2016). Prognostic factors for the feasibility of chemotherapy and the Geriatric Prognostic Index (GPI) as risk profile for mortality before chemotherapy in the elderly. Acta Oncol.

[33] Kalsi T, Babic-Illman G, Ross PJ, Maisey NR, Hughes S, Fields P (2015). The impact of comprehensive geriatric assessment interventions on tolerance to chemotherapy in older people. Br J Cancer.

[34] Corre R, Greillier L, Le Caër H, Audigier-Valette C, Baize N, Bérard H (2016). Use of a comprehensive geriatric assessment for the management of elderly patients with advanced non-small-cell lung cancer: the phase III randomized ESOGIA-GFPC-GECP 08–02 study. J Clin Oncol.

[35] Extermann M, Boler I, Reich RR, Lyman GH, Brown RH, DeFelice J (2012). Predicting the risk of chemotherapy toxicity in older patients: the Chemotherapy Risk Assessment Scale for High-Age Patients (CRASH) score. Cancer.

[36] Hurria A, Mohile S, Gajra A, Klepin H, Muss H, Chapman A (2016). Validation of a prediction tool for chemotherapy toxicity in older adults with cancer. J Clin Oncol.

[37] Rao A, Cohen HJ (2004). Symptom management in the elderly cancer patient: fatigue, pain, and depression. J Natl Cancer Inst Monogr.

[38] Cataldo JK, Paul S, Cooper B, Skerman H, Alexander K, Aouizerat B (2013). Differences in the symptom experience of older versus younger oncology outpatients: a cross-sectional study. BMC Cancer.

[39] Loh KP, McHugh C, Mohile SG, Mustian K, Flannery M, Klepin H (2018). Using information technology in the assessment and monitoring of geriatric oncology patients. Curr Oncol Rep.

[40] Jensen RE, Snyder CF, Abernethy AP, Basch E, Potosky AL, Roberts AC (2014). Review of electronic patient-reported outcomes systems used in cancer clinical care. J Oncol Pract.

[41] Basch E, Deal AM, Kris MG, Scher HI, Hudis CA, Sabbatini P (2016). Symptom monitoring with patient-reported outcomes during routine cancer treatment: a randomized controlled trial. J Clin Oncol.

[42] Denis F, Lethrosne C, Pourel N, Molinier O, Pointreau Y, Domont J (2017). Randomized trial comparing a web-mediated follow-up with routine surveillance in lung cancer patients. J Natl Cancer Inst.

[43] Nightingale G, Pizzi LT, Barlow A, Barlow B, Jacisin T, McGuire M (2018). The prevalence of major drug-drug interactions in older adults with cancer and the role of clinical decision support software. J Geriatr Oncol.

[44] Maggiore RJ, Gross CP, Hurria A (2010). Polypharmacy in older adults with cancer. Oncologist.

[45] Steinman M, Hanlon J (2010). Managing medications in clinically complex elders: "There's got to be a happy medium". JAMA.

[46] Scripture CD, Figg WD (2006). Drug interactions in cancer therapy. Nat Rev Cancer.

[47] Ferri N, Bellosta S, Baldessin L, Boccia D, Racagni G, Corsini A (2016). Pharmacokinetics interactions of monoclonal antibodies. Pharmacol Res.

[48] Serra López-Matencio J, Martínez Nieto C, Morell Baladrón A, Castañeda S (2018). Drug interactions of monoclonal antibodies-clinical perspective. J Immunol Sci.

[49] Sokol KC, Knudsen JF, Li MM (2007). Polypharmacy in older oncology patients and the need for an interdisciplinary approach to side-effect management. J Clin Pharm Ther.

[50] Chen L, Cheung WY (2014). Potential drug interactions in patients with a history of cancer. Curr Oncol.

[51] Presley CJ, Dotan E, Soto-Perez-de-Celis E, Jatoi A, Mohile SG, Won E (2016). Gaps in nutritional research among older adults with cancer. J Geriatr Oncol.

[52] Vigano A, Kasvis P, Di Tomasso J, Gillis C, Kilgour R, Carli F (2017). Pearls of optimizing nutrition and physical performance of older adults undergoing cancer therapy. J Geriatr Oncol.

[53] Wischmeyer PE, Carli F, Evans DC, Guilbert S, Kozar R, Pryor A (2018). American society for enhanced recovery and perioperative quality initiative joint consensus statement on nutrition screening and therapy within a surgical enhanced recovery pathway. Anesth Analg.

[54] Deutz NE, Matheson EM, Matarese LE, Luo M, Baggs GE, Nelson JL (2016). Readmission and mortality in malnourished, older, hospitalized adults treated with a specialized oral nutritional supplement: a randomized clinical trial. Clin Nutr.

[55] Mohile SG, Fan L, Reeve E, Jean-Pierre P, Mustian K, Peppone L (2011). Association of cancer with geriatric syndromes in older Medicare beneficiaries. J Clin Oncol.

[56] Wildes TM, Dua P, Fowler SA, Miller JP, Carpenter CR, Avidan MS (2015). Systematic review of falls in older adults with cancer. J Geriatr Oncol.

[57] Ward PR, Wong MD, Moore R, Naeim A (2014). Fall-related injuries in elderly cancer patients treated with neurotoxic chemotherapy: a retrospective cohort study. J Geriatr Oncol.

[58] Mohile SG, Dale W, Somerfield MR, Schonberg MA, Boyd CM, Burhenn PS (2018). Practical assessment and management of vulnerabilities in older patients receiving chemotherapy: ASCO guideline for geriatric oncology. J Clin Oncol.

[59] Wildes TM, Ruwe AP, Fournier C, Gao F, Carson KR, Piccirillo JF (2013). Geriatric assessment is associated with completion of chemotherapy, toxicity, and survival in older adults with cancer. J Geriatr Oncol.

[60] Magnuson A, Sattar S, Nightingale G, Saracino R, Skonecki E, Trevino KM (2019). A practical guide to geriatric syndromes in older adults with cancer: a focus on falls, cognition, polypharmacy, and depression. Am Soc Clin Oncol Educ Book.

[61] Jayani R, Hurria A (2012). Caregivers of older adults with cancer. Semin Oncol Nurs.

[62] Kehoe LA, Xu H, Duberstein P, Loh KP, Culakova E, Canin B (2019). Quality of life of caregivers of older patients with advanced cancer. J Am Geriatr Soc.

[63] Chau I, Norman AR, Cunningham D, Waters JS, Oates J, Ross PJ (2004). Multivariate prognostic factor analysis in locally advanced and metastatic esophago-gastric cancer–pooled analysis from three multicenter, randomized, controlled trials using individual patient data. J Clin Oncol.

[64] Takashima A, Shitara K, Fujitani K, Koeda K, Hara H, Nakayama N (2019). Peritoneal metastasis as a predictive factor for nab-paclitaxel in patients with pretreated advanced gastric cancer: an exploratory analysis of the phase III ABSOLUTE trial. Gastric Cancer.

[65] Hamamoto Y (2015). Complications in advanced or recurrent gastric cancer patients with peritoneal metastasis during and after palliative systemic chemotherapy. Mol Clin Oncol.

[66] Kitayama J, Ishigami H, Yamaguchi H, Sakuma Y, Horie H, Hosoya Y (2018). Treatment of patients with peritoneal metastases from gastric cancer. Ann Gastroenterol Surg.

[67] Arai H, Iwasa S, Boku N, Kawahira M, Yasui H, Masuishi T (2019). Fluoropyrimidine with or without platinum as first-line chemotherapy in patients with advanced gastric cancer and severe peritoneal metastasis: a multicenter retrospective study. BMC Cancer.

[68] Kobayashi M, Sakamoto J, Namikawa T, Okamoto K, Okabayashi T, Ichikawa K (2006). Pharmacokinetic study of paclitaxel in malignant ascites from advanced gastric cancer patients. World J Gastroenterol.

[69] Kochi M, Fujii M, Kanamori N, Kaiga T, Okubo R, Mihara Y (2011). Pharmacokinetics of oxaliplatin in gastrointestinal cancer patients with malignant ascites. J Chemother.

[70] Masuishi T, Kadowaki S, Kondo M, Komori A, Sugiyama K, Mitani S (2017). FOLFOX as first-line therapy for gastric cancer with severe peritoneal metastasis. Anticancer Res.

[71] Nakajima TE, Yamaguchi K, Boku N, Hyodo I, Mizusawa J, Hara H, et al. Randomized phase II/III study of 5-fluorouracil/l-leucovorin versus 5-fluorouracil/l-leucovorin plus paclitaxel administered to patients with severe peritoneal metastases of gastric cancer (JCOG1108/WJOG7312G). Gastric Cancer. 2020. 10.1007/s10120-020-01043-x. 10.1007/s10120-020-01043-x 32036492

[72] Saif M, Siddiqui I, Sohail M (2009). Management of ascites due to gastrointestinal malignancy. Ann Saudi Med.

[73] Maeda H, Kobayashi M, Sakamoto J (2015). Evaluation and treatment of malignant ascites secondary to gastric cancer. World J Gastroenterol.

[74] Hasegawa H, Fujitani K, Nakazuru S, Hirao M, Yamamoto K, Mita E (2014). Optimal treatment change criteria for advanced gastric cancer with non-measurable peritoneal metastasis: symptom/tumor marker-based versus CT-based. Anticancer Res.

[75] Stukan M (2017). Drainage of malignant ascites: patient selection and perspectives. Cancer Manag Res.

[76] Ito T, Hanafusa N (2017). CART: cell-free and concentrated ascites reinfusion therapy against malignancy-related ascites. Transfus Apher Sci.

[77] Nie R, Yuan S, Chen S, Chen X, Chen Y, Zhu B (2016). Prognostic nutritional index is an independent prognostic factor for gastric cancer patients with peritoneal dissemination. Chin J Cancer Res.

[78] Hamauchi S, Furuse J, Takano T, Munemoto Y, Furuya K, Baba H (2019). A multicenter, open-label, single-arm study of anamorelin (ONO-7643) in advanced gastrointestinal cancer patients with cancer cachexia. Cancer.

